# Coffee and its waste repel gravid *Aedes albopictus* females and inhibit the development of their embryos

**DOI:** 10.1186/s13071-015-0874-6

**Published:** 2015-05-14

**Authors:** Tomomitsu Satho, Hamady Dieng, Muhammad Hishamuddin Itam Ahmad, Salbiah Binti Ellias, Ahmad Abu Hassan, Fatimah Abang, Idris Abd Ghani, Fumio Miake, Hamdan Ahmad, Yuki Fukumitsu, Wan Fatma Zuharah, Abdul Hafiz Ab Majid, Nur Faeza Abu Kassim, Nur Aida Hashim, Olaide Olawunmi Ajibola, Fatima Abdulla Al-Khayyat, Cirilo Nolasco-Hipolito

**Affiliations:** Faculty of Pharmaceutical Sciences, Fukuoka University, Fukuoka, Japan; Institute of Biodiversity and Environmental Conservation, Universiti Malaysia Sarawak, Kota Samarahan, Malaysia; School of Biological Sciences, Universiti Sains Malaysia, Penang, Malaysia; Faculty of Resource Science and Technology, Universiti Malaysia Sarawak, Kuching, Malaysia; Faculty of Science and Technology, Universiti Kebangsaan Malaysia, Bangi, 43600 Selangor, Malaysia; School of Food Science and Technology, Universiti Malaysia Terengganu, Kuala Terengganu, Malaysia; Department of Biological and Environmental Sciences, College of Arts and Sciences, Qatar University, Doha, Qatar

**Keywords:** *Aedes albopictus*, Coffee, Oviposition, Embryonation, Egg hatching

## Abstract

**Background:**

Dengue is a prevalent arboviral disease and the development of insecticide resistance among its vectors impedes endeavors to control it. Coffee is drunk by millions of people daily worldwide, which is associated with the discarding of large amounts of waste. Coffee and its waste contain large amounts of chemicals many of which are highly toxic and none of which have a history of resistance in mosquitoes. Once in solution, coffee is brownish in colour, resembling leaf infusion, which is highly attractive to gravid mosquitoes. To anticipate the environmental issues related to the increasing popularity of coffee as a drink, and also to combat insecticide resistance, we explored the deterrence potentials of coffee leachates against the ovipositing and embryonic stages of the dengue vector, *Aedes albopictus*.

**Methods:**

In a series of choice, no-choice, and embryo toxicity bioassays, we examined changes in the ovipositional behaviours and larval eclosion of *Ae. albopictus* in response to coffee extracts at different concentrations.

**Results:**

Oviposition responses were extremely low when ovicups holding highly concentrated extract (HCE) of coffee were the only oviposition sites. Gravid females retained increased numbers of mature eggs until 5 days post-blood feeding. When provided an opportunity to oviposit in cups containing coffee extracts and with water, egg deposition occurred at lower rates in those containing coffee, and HCE cups were far less attractive to females than those containing water only. Females that successfully developed in a coffee environment preferentially oviposited in such cups when in competition with preferred oviposition sites (water cups), but this trait did not continue into the fourth generation. Larval eclosion occurred at lower rates among eggs that matured in a coffee environment, especially among those that were maintained on HCE-moistened substrates.

**Conclusions:**

The observations of the present study indicate a pronounced vulnerability of *Ae. albopictus* to the presence of coffee in its habitats during the early phases of its life cycle. The observations that coffee repels gravid females and inhibits larval eclosion provide novel possibilities in the search for novel oviposition deterrents and anti-larval eclosion agents against dengue vectors.

## Background

The mosquito *Aedes albopictus* originated from Asian forests [1], but has spread around the world to become a serious threat to public health in many countries [2]. This mosquito is a proven vector of many viruses [3], but is best known as a dengue vector [4]. This disease is by far the most prevalent human arboviral infection worldwide [5], with 50 – 100 million dengue infections worldwide annually, thousands of deaths [5], and more than 2.5 billion people at risk [6]. Formerly regarded as a secondary vector, *Ae. albopictus* has joined *Aedes aegypti* in many parts of the world [7] and has been suggested to have played a role in many recent outbreaks of not only dengue [8,9] but also chikungunya [10].

The first and main line of control against dengue vectors is the use of insecticides [6]. However, such vectors have acquired resistance to the main families of insecticides currently used for vector control around the world [11]. Most control strategies focus on mosquito behaviours [12]. For disease transmission, a female mosquito must complete three actions: take up the pathogen via blood feeding, rest to digest the blood meal, and oviposition. Although each of these behaviours can be targeted, aiming at the resting population is difficult even with skilled workers [13]. In contrast, strategies targeting gravid females, which are more likely to be infected (i.e., to have fed on a blood meal at least once), will directly reduce the incidence of disease as they will not necessarily transmit the pathogen. Such approaches are suitable for *Ae. albopictus*, which is capable of transovarial transmission [14].

The successful spread of *Ae. albopictus* across the globe is a direct consequence of its oviposition behaviour—transport of eggs in used tires or lucky bamboo [15]. Female *Ae. albopictus* mosquitoes have been shown to oviposit in many human-made containers [16], and they are able to identify high-quality egg-laying sites [17]. They preferentially oviposit on sites that provide sufficient litter-based resources [18] because larval performance is responsive to the presence/absence of leaf litter. There has been a great deal of research regarding the visual parameters of attraction to female mosquitoes, but most of these studies addressed odorants [19]. For dengue vectors that are diurnal, however, visual cues are undoubtedly crucial in container choice for egg deposition [20,19]. It has been suggested that taking oviposition into account when designing dengue vector control programmes can lead to more effective prevention [21]. This natural behaviour influences survival, growth, population distribution, and abundance [22]. Himeidan and colleagues [23] argued for the need to lure females to lethal traps to increase their exposure to insecticides. Many lethal trapping trials have been performed around the world [24,25]. Despite reductions in population size, few of these programmes have achieved elimination, and most were associated with low trap attractiveness [26]. Most of these trials were carried out using strips treated with synthetic insecticide and were performed without addressing the influences of the physical characteristics of the aquatic media, such as colour, on oviposition choice. A recent study examined the effects of water surface area, but the authors did not address lethal ovitrapping and ignored the colour of the water. This aspect of the aquatic media is important as *Aedes* mosquitoes, particularly *Ae. albopictus*, are generally active during the day and rely mostly on optical cues [27]. Another drawback of these apparatuses is their inability to compete with other potential breeding sites [24,28].

In nature, *Aedes* larvae feed on microorganisms present on leaf surfaces [29] or by directly ingesting particulate matter [30]. After falling into an artificial container, leaf substrate undergoes decomposition, which results in the release of many compounds causing the water to become brownish in colour [31]. Dieng and co-workers [32] examined the effects of water coloured to different extents by to the decomposition of cigarette butts (CBs) on oviposition responses of *Ae. albopictus* and observed significantly more egg accumulation in dark brown water than in tap water. This effect was attributed to the presence of leachates, which produced visual stimuli regarded by females as good signals for egg deposition. Similar to CBs, coffee can also enter into solution by dissolution [33,34]. This beverage, produced from roasted seeds of *Coffea* plants [35], is one of the most widely consumed drinks in the world [36]. Its increased intake has been accompanied by the discarding of huge amounts of waste [37] into the environment. For example, several million tons of coffee grounds are discarded annually in the UK [38]. In Hong Kong, Starbucks produces about 5000 tons of used coffee grounds per year [39]. Global coffee bean exports and consumption have been forecast to reach record levels in the coming years [40]. There are over 1000 chemical compounds in coffee [41]. A variety of chemicals occur naturally in the raw coffee bean [42,43] and about 950 more new compounds are formed after roasting [44]. Coffee may also contain chemical insecticides used during the planting process [45]. The storage conditions [46,47] and roasting methods [48] influence the chemical composition of coffee. Many of these compounds are highly toxic and have negative effects on animals [49,50]. For example, caffeine has been reported to have adverse effects on the brains of rodents and monkeys [51]; exposure to coffee causes teratogenic and neurodevelopmental problems in some small mammals [52]; consumption of coffee pulp causes a reduction of egg hatchability and death in hens [53], and body weight loss in chickens [53] and cattle [54]; and caffeine alters gender balance in hamsters [55] and hinders fetal development of *Rattus norvegicus* [56].

There are many reasons for optimism that coffee may be useful in mosquito control. Some *Coffea* plants are naturally resistant to insect attack [57]. Roasting coffee beans produces compounds mutagenic to bacteria [58]. Coffee has antibacterial and antiviral properties [59]. Caffeine impedes the web-building activity of spiders [49], kills certain insects [60,61], and inhibits feeding in flies and beetles [62]. Coffee also decreases the reproductive capacity of mosquitoes [63], egg development in flies [64,65], and is highly lethal to *Ochlerotatus notoscriptus* [66]. The increased diversity in structural and processing toxicities [44,45] may impose variations in acting toxicants and variability in modes of action and complex potential formulations, a strategy recognized as a potential modifier in insecticide resistance management [67]. None of the chemicals in coffee has a history of resistance in insects. In addition, reusing coffee waste to control insect pests will help reduce its incidence in the environment. Here, we examined whether *Ae. albopictus* alters its ovipositional responses in response to the presence of fresh and used coffee in its habitats.

## Methods

### Colony

The *Ae. albopictus* mosquitoes used here were from a colony kept under controlled conditions [temperature 29 ± 3.0°C, relative humidity 75% ± 5% RH, and photoperiod of 13:10 (light:dark) with 1 hour of dusk] at the insectary of the School of Biological Sciences, University Sains Malaysia. 120 to 150 larvae were raised in metallic enamelware pans, 12 cm in diameter and 2 cm in depth, holding 1 L of dechlorinated water. They were fed every 2 days with a mixture consisting of dog biscuits, beef liver, yeast, and milk powder in a weight ratio of 2:1:1:1, as reported elsewhere [68]. The food was supplied as a suspension (0.15 g of larval food in 4 mL of water) in increasing amounts (day 1: 3 drops; day 3: 5 drops; and day 5: 7 drops) using pipettes [69]. The rearing medium was replaced with fresh water before the third food supply). Pupae were held in plastic cups (250-mL capacity) and transferred into standard 30 × 30 × 30 cm rearing cages. Adults were given continuous access to 10% sucrose solution. Females were blood-fed on restrained mice about 4–5 days after emergence. Oviposition devices (plastic containers 11.5 cm in diameter and 6.2 cm in depth, bordered interiorly with a piece of filter paper as an oviposition substrate) were placed within cages for egg collection 3 days after blood intake. Eggs were air-dried under insectarium conditions and kept in small plastic vessels for utilization as an egg bank for colony continuation.

### Production of experimental mosquitoes

To obtain experimental virgin males and females, egg samples from the egg bank were hatched in dechlorinated water, and larvae 24 hours old were reared at a density of 150 per enamelware pan of the same dimensions as those used in colony maintenance filled with 1 L of dechlorinated water in ten replicates. The diet was provided following a slight modification of the procedure reported elsewhere [70,71] as suspension [0.15 g of mixture of dog biscuits, beef liver, yeast, and milk powder (2:1:1:1) in 5 mL of dechlorinated water] dispensed according to Juliano and Gravel [69]. Larval feeding was standardized and the timetable and quantities of food given were as follows: 3, 6, 6, 6, 6, and 6 mL of larval food suspension on days 1, 3, 5, 7, 9, and 11, respectively. Rearing media was replaced with fresh water prior to supplying food on days 3 and 7. To ensure sex separation, pupae were kept in individual 1.5-mL Eppendorf tubes holding 0.05 mL of dechlorinated water. Pupation was checked on a daily basis, and the sex of the mosquitoes was determined upon pupal transformation to adults. Males were grouped in standard cages the same as those used in colony maintenance marked “M” and females were pooled in cages tagged “F”. All adults had continuous access to 10% sucrose solution through a cotton pad soaked with 10% sucrose solution, held in a 250-mL plastic container that was replaced every 2 days.

### Experimental coffee strain and tested experimental extracts

*Coffea canephora* from Kedah State, Malaysia, was used in this study. Dried roasted beans were ground using a mortar and pestle and the resulting fine powder was kept in sealed plastic containers and stored at –18°C. Ten replicates of 4.7 g of powdered roasted coffee were placed into individual 250-mL glass vials containing 150 mL of boiled water. After one hour of soaking, the mixtures were sieved with fine mesh mosquito netting and the resulting infusions were pooled and referred to as Highly Concentrated Extract (HCE). Infusions obtained by soaking of the ground remnants from the sieving of 4.7 g, 2.35, and 1.17 g of powdered roasted coffee for the same period in the same volume (only for 2.35 and 1.17 g) of hot water were designated as Used Highly Concentrated Extract (UHCE), Moderately Concentrated Extract (MCE), and Low Concentration Extract (LCE), respectively (Table 1).

**Table 1:** **The experimental extracts used in this study**

**Solution**	**Procedure for production**
HCE	► 4.7 g of roasted coffee was allowed to completely dissolve in 150 mL of deionized water for 1 h; the solution was filtered using fine mesh mosquito netting
UHCE	► 4.7 g of used roasted coffee was allowed to completely dissolve in 75 mL of deionized water for 1 h; the solution was filtered using fine mesh mosquito netting
MCE	► 2.35 g of roasted coffee was allowed to completely dissolve in 150 mL of deionized water for 1 h; the solution was filtered using fine mesh mosquito netting
LCE	► 1.35 g of roasted coffee was allowed to completely dissolve in 150 mL of deionized water for 1 h; the solution was filtered using fine mesh mosquito netting
WAa, WAb, or WAc	► 100 mL of cool boiled water

### Experiments

**Figure 1:**
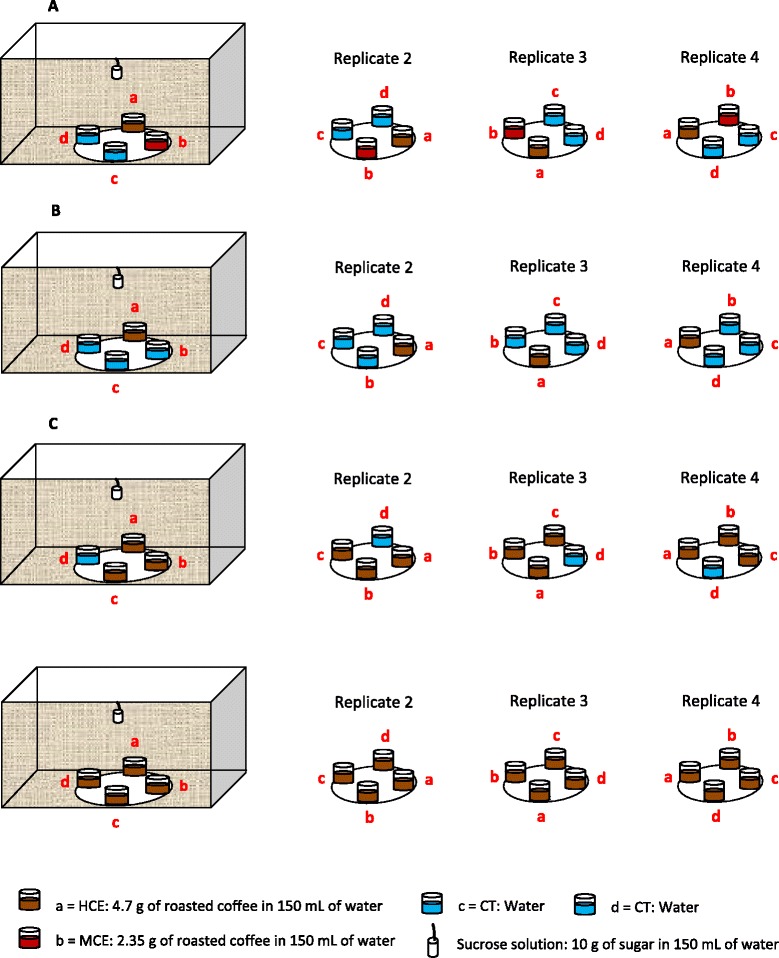
Oviposition bioassay design. The cups were placed on the dish, which was positioned at the bottom center of the cage. Cups were placed such that each was at an identical distance from the adjacent cup. **A**: The four oviposition cups contained one of the following media: (i) HCE, (ii) MCE, (iii) cooled boiled water (WAa), or (iv) cooled boiled water (WAb); **B**: The four oviposition cups contained one of the following media: (i) HCE, (ii) cooled boiled water (WAa), (iii) cooled boiled water (WAb), or (iv) cooled boiled water (WAc); **C**: The four oviposition cups contained one of the following media: (i) HCEa, (ii) HCEb, (iii) HCEc, and (iv) cooled boiled water (WA).

The oviposition device used in all experiments was similar to that of Dieng *et al.* [17] and consisted of a circular white plastic dish 12 cm in diameter and four plastic ice cream cups 9 cm in depth with lower and upper diameters of 5 cm and 7.5 cm, respectively. Each cup was interiorly lined with filter paper as an oviposition substrate. The cups were placed on the dish, which was positioned at the bottom centre of the cage. Cups were placed such that each was at an identical distance from the adjacent cup (Figure 1). Each cup holding coffee extract and the control cups (tap water) were replicated four times and a replicate coincided with one position of the four cups on the circular plastic. For each replicate of the experiment, a new batch of adult mosquitoes and new coffee extract were used. All females used were starved for 12 hours before bioassay. In Experiment 2, the oviposition tubes used consisted of an acrylic tube (7.5 cm in depth and 3.2 cm in diameter, the interior of which was covered with a piece of filter paper as an egg deposition substrate, similar to those used previously [17]. Briefly, a 1.5-mL Eppendorf tube with the lower bottom surface removed was fixed to the tube cover and obstructed with a cotton pad imbibed with 10% sucrose solution to feed the tested female.

### Oviposition activity of *Ae. albopictus* to coffee media in competition with water

This bioassay was performed to examine the oviposition preferences of *Ae. albopictus* females in the presence of four egg deposition sites, including those containing coffee extracts. Twenty-five males (2- 5 days old) were placed in a cage (30 × 30 × 30 cm) with access to 10% sucrose solution. Sixty starved females (3–4 days old) were added to the cage. The sucrose solution was removed from the cage and the adults were allowed to acclimatize to the new environment. After 30 minutes of acclimatization to the cage environment, one restrained white mouse was placed inside the cages. After 1 hour of blood feeding, the mouse was removed from the cage. Three days after blood feeding, 15 gravid females were transferred to another cage containing the oviposition device. They were given access to oviposition cups containing 200 mL of one of the following media: (i) HCE, (ii) MCE, (iii) cooled boiled water (WAa), or (iv) cooled boiled water (WAb). To determine whether gravid *Ae. albopictus* females are attracted to coffee-treated water in the presence of increased oviposition opportunities in water, 25 sugar-fed males (2-5 days old) were allowed to cohabit with 60 starved females (3- 4 days old) in the same cage type as described above. Adults were allowed to acclimatize to the new environment for 30 minutes, after which the females were given a chance to feed on blood by placing an immobilized mouse inside the cage. One hour later, the blood source was removed and the females were permitted to digest the meals in the presence of a 10% sucrose solution. After 3 days of blood digestion, 15 females were moved to a new cage holding an oviposition device with the four cups each filled with 200 mL of one of the following solutions: (i) HCE, (ii) cooled boiled water (WAa), (iii) cooled boiled water (WAb), or (iv) cooled boiled water (WAc). To determine whether gravid *Ae. albopictus* females are enticed by coffee-treated water in the presence of reduced oviposition opportunities in water, the same procedures and numbers of males and females as reported in Experiment 2 were repeated but with the following modifications: the 15 females were given opportunities to lay eggs in the following media: (i) HCEa, (ii) HCEb, (iii) HCEc, and (iv) cooled boiled water (WA).

### Coffee exposure and egg retention

The low levels of oviposition responses observed in cups containing coffee extracts prompted us to examine whether this species exhibits egg retention in response to the presence of coffee in potential egg deposition sites. An oviposition bioassay was performed as described in the first experiment in Study 1 above, but with two modifications: a) 12 females were given a chance to oviposit in four cups all of which contained HCE; b) the volume of extract in each of the cups was 100 mL.

### Effects of exposure of eggs to coffee during maturation on hatching success

To examine whether eclosion of *Ae. albopictus* larvae is affected by exposure to coffee, 24 starved females (3- 4 days old) and 12 sugar-fed males (2- 5 days old) were encaged and allowed to cohabit. Following a 30-minute acclimatization period, females were provided with a blood meal as described above. All fully blood-fed females and sugar-fed males were transferred into a new cage where they had access to a sugar diet (10% sucrose solution). After 3 days of blood meal digestion, 30 gravid females were divided into three groups of ten females each. Each group of females was placed in an oviposition tube and assigned to one of the following oviposition conditions: (i) oviposition substrate imbibed with HCE, (ii) oviposition substrate imbibed with UHCE, and (iii) oviposition substrate imbibed with WA. After a 3-day oviposition period, females were removed from cages and egg deposition was monitored by examining the oviposition substrates under a dissecting microscope. Eggs were then allowed to dry and embryonate for 3 days on their respective substrates under insectarium conditions as described previously [72]. All dried eggs that achieved development on each of the oviposition substrates imbibed with (i) HCE, (ii) UHCE, or (iii) WA were placed in plastic containers (250-mL capacity), where they were flooded with 50 mL of hatching solution similar to that described previously [17] consisting of 2 mL of 2-day-old tap water added to two droplets of dried yeast solution (0.003 g/100 mL). Larval eclosion success was noted 24 hours after flooding.

### Larval exposure to coffee and ovipositional response to coffee

This study was performed to determine whether females derived from larvae that developed in an environment containing coffee avoid laying eggs in oviposition sites containing coffee extract. A total of 150 newly hatched larvae (24 hours old) were placed in a 250-mL capacity plastic container with 200 mL of HCE supplemented with 3 mL of larval food suspension. After 3 hours in the HCE medium, larvae were collected, rinsed with distilled water, and transferred to an enamelware pan (12 cm in diameter × 2 cm deep) containing 500 mL of MCE. Seven other cohorts, each with 150 newly hatched larvae, were treated as described above and were moved to four other pans with the same amount of MCE. Larvae were fed according to the timetable and amounts used in the production of experimental mosquitoes. Pupation was inspected daily, and pupated individuals were singly transferred into 1.5-mL Eppendorf tubes filled with 0.05 mL of dechlorinated water. As in the production of experimental mosquitoes, males and females were separately pooled in cages to avoid fertilization before the experiments. Both cages held a sugar source consisting of a glass tube (2 × 8 cm) filled with 10% sucrose solution connected to a cotton pad. Four days after emergence, females that were starved for 12 hours were transferred into the cage holding males. A restrained mouse was placed within the cage to serve as a blood meal source for 30 minutes. Fully engorged females were immediately moved to a new cage and given access to the sugar diet. On day 3 after blood feeding, 60 females were divided into four groups of 15 individuals each given a choice to lay eggs in four cups filled with 200 mL of (i) HCE, (ii) MCE, (iii) WAa, or (iv) WAb, following the clockwise replication design shown in Figure 1. The next day, all remaining males and females were pooled into one cage, maintained on sugar, and females were allowed to take a blood meal from two restrained mice for 1 hour. Three days after blood meal uptake, four acrylic tubes (7.5 cm in depth, 3.2 cm in diameter, lined with a piece of filter paper as oviposition substrate) were placed inside the cage for egg collection. Eggs were allowed to dry on the filter paper substrates under laboratory conditions as reported elsewhere [72]. After a 3 day-drying period, the filter papers with dried eggs were flooded in a 250-mL plastic container with 25 mL of hatching medium similar to that described earlier [17]. These F2 larvae were fed the same as their larval parents and development was followed until their F3 counterparts emerged as adults. The same rearing procedures and adult treatments were carried out until sufficient numbers of F4 adults were obtained. As described for their adult parents, 60 F4 females were divided into four groups of 15 females each. Each group was given access to oviposition cups filled with 100 mL of (i) HCE, (ii) MCE, (iii) WAa, or (iv) WAb, with replicates as described above.

### Data collection

In all oviposition bioassays, the oviposition period was 3 days. The numbers of eggs laid on oviposition substrates, those deposited on the edges, and on the bottom of oviposition devices (cups or glass tubes) were counted under a dissecting microscope (Meiji EMZ; Meiji Techno Co., Ltd., Tokyo, Japan). The means of these numbers were used as measures of oviposition response. Oviposition responses were also compared based on differences in percentage of eggs laid. We considered the percentage of eggs laid in a given medium as the total number of eggs deposited during a given bioassay (all replicates) divided by the total number of eggs deposited in all media. In the egg retention study, the total number of eggs retained per female in a given bioassay was calculated as the total number of eggs laid (all replicates) divided by the number of females used. In the larval eclosion study, the number of eggs that hatched was determined after 24 hours of flooding by counting the number of first instar larvae. These numbers were utilized to compute egg hatching rate as the number of hatched eggs divided by the total number of eggs (unhatched + hatched) flooded × 100.

### Statistical analysis

The differences in oviposition and egg hatching responses between the different media and female types were compared by analysis of variance (ANOVA) using the Systat v.11 statistical software package [73] and Tukey’s Honestly Significant Difference test where necessary. In all analyses, *P* < 0.05 was taken to indicate statistical significance.

### Ethical approval

This study was carried out in accordance with the principles expressed in the Declaration of Helsinki. The study was approved by the Biological Research Ethics Committee at University Sains Malaysia.

## Results

### Oviposition preferences to coffee media in different competition levels with water-containing ovicups

**Figure 2:**
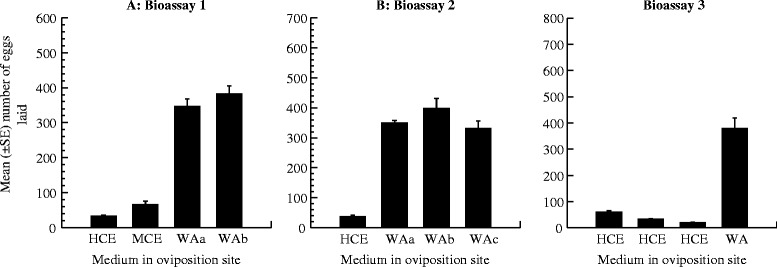
Responses of gravid *Ae. albopictus* females when given choice to oviposit in four cups containing coffee media at different levels with water ovicups. **A**: Oviposition sites: (i) HCE, (ii) MCE, (iii) cooled boiled water (WAa), and (iv) cooled boiled water (WAb); **B**: Oviposition sites: (i) HCE, (ii) cooled boiled water (WAa), (iii) cooled boiled water (WAb), and (iv) cooled boiled water (WAc); **C**: Oviposition sites: (i) HCEa, (ii) HCEb, (iii) HCEc, and (iv) cooled boiled water (WA).

When given equal opportunities to oviposit in two cups containing coffee extracts and two others with water, eggs were deposited in all four cups, but egg deposition differed considerably with cup medium. A total of 3306 eggs were laid by 60 females, of which 88.23% (2906/33306) were oviposited in cups containing water and 11.77% (389/3306) in cups containing coffee extracts. Egg deposition was highest in WAb cups (383.00 ± 70.86) and decreased in the order WAa (364.25 ± 65.29) > HCE (65.25 ± 31.12) > MCE (32.00 ± 12.12) (ANOVA DF = 3, P < 0.001) (Figure 2A). When there were more oviposition opportunities in cups containing water, eggs were deposited in all four cups. Of the 4462 eggs laid by the 60 females, 3.34% (149/4462) were deposited in cups containing HCE and 96.66% (4313/4462) in cups containing water (WAa: 350 ± 24.23 eggs; WAb: 389 ± 102.781 eggs; WAc: 330.25 ± 80.02 eggs). Oviposition was lowest in the presence of coffee leachates (ANOVA, DF = 3, P = 0.010) (Figure 2B). When there were more oviposition options in cups containing coffee extract, egg deposition was observed in all four cups. The total of 1966 eggs laid consisted of 1514 in cups holding water and 452 in the three cups containing HCE (HCEa: 60.25 ± 15.72 eggs; HCEb: 32.75 ± 8.53 eggs; and HCEc: 20.00 ± 4.38 eggs), representing 77% and 23% of the total, respectively. Significantly, more eggs were laid in the single cup containing water than in those with HCE (ANOVA = 7.319, DF = 3, P = 0.005) (Figure 2C). Overall, cups containing coffee extracts, especially those with the greatest coffee concentration, were significantly less attractive to ovipositing *Ae. albopictus* than cups containing water.

### Oviposition deterrence

**Figure 3:**
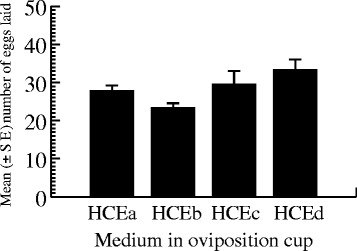
Responses of gravid *Ae. albopictus* females when only given the option to oviposit in four cups containing highly concentrated coffee extract (HCE).

Figure 3 shows the oviposition responses of *Ae. albopictus* when provided only with cups containing coffee extract as egg deposition sites. Females oviposited in all cups with a mean egg deposition rate of 28.43 ± 4.44 eggs per cup (HCEa: 27.75 ± 5.95 eggs, range: 16 – 44 eggs; HCEb: 23.25 eggs ± 5.20, range: 15 – 38 eggs; HCEc: 29.50 ± 13.86 eggs, range: 14 –71 eggs; and HCEd: 33.25 ± 11.09 eggs, range: 18 – 65 eggs). There were no significant differences in egg number between the four cups (ANOVA = 0.183, DF = 3, P = 0.906).

### Comparison of oviposition responses in relation to oviposition opportunities and egg retention

When given identical options to oviposit in cups containing coffee extracts (HCE and MCE) and two others containing water (WAa and WAb), the mean number of eggs laid per female was 55.1 (3306/15 females). These values were 74.36 (4462/15 females), 32.76 (1966/15 females), and 9.47 eggs (455/12 females) for females given (i) more oviposition chances in cups containing water (HCE, WAa, WAb, WAc), (ii) more oviposition opportunities in cups containing coffee extract (HCEa, HCEb, HCEc, WA), and (iii) only cups containing HCE as egg deposition sites (HCEa, HCEb, HCEc, HCEd), respectively. The total numbers of eggs laid in the first three bioassays were 7.2, 9.8, and 4.32 times higher than that when four cups containing HCE were the only egg deposition sites, respectively (Table 2). In this latter bioassay, a total of 576 eggs were collected from ovaries of 12 females, 5 days after blood meal feeding, indicating that 55.86% (576/1031) of the eggs were retained by the experimental female cohort at an average of 48 eggs (576/12 females) per female.

**Table 2:** **Comparison of oviposition responses in relation to oviposition opportunities and egg retention**

**Study**	**The four oviposition cup**	**Total number of eggs**				**Total number of eggs laid**	**Total number of females**
		**Replicate 1**	**Replicate 2**	**Replicate 3**	**Replicate 4**		
One	• HCE, MCE, WAa WAb	796	1027	1009	474	3306	15
	• HCE, WAa, WAb, WAc	688	1201	1567	1006	4462	15
	• HCEa, HCEb, HCEc, WA	214	531	440	334	1966	15
Two	• HCEa, HCEb, HCEc, HCEd	97	131	86	141	455	12

### Larval eclosion responses

**Figure 4:**
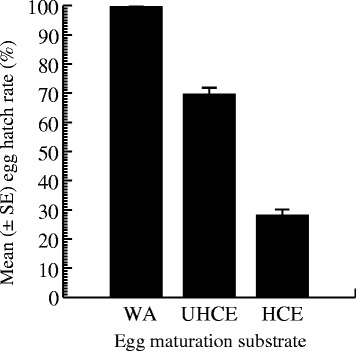
Hatching rates of *Ae albopictus* eggs (mean ± SE) that matured on substrates moistened with tap water (WA controls) and substrates moistened with different amounts of coffee extract.

Egg hatching responses varied significantly with medium type (ANOVA = 39.67, DF = 2, P < 0.001). The mean hatching rate of eggs that matured in the water-moistened environment (99.54 ± 0.45%, range: 95.45% - 100%) was significantly higher than that of eggs that were maintained in the UHCE environment (69.60 ± 7.43%, range: 25.53% – 90%) (Tukey HSD, P = 0.003), which in turn was lower than that recorded among eggs that developed in the HCE environment (28.10 ± 6.47, range: 0% – 65%) (Tukey HSD, P < 0.001) (Figure 4).

### Oviposition responses in relation to history of coffee exposure in the larval stage

**Figure 5:**
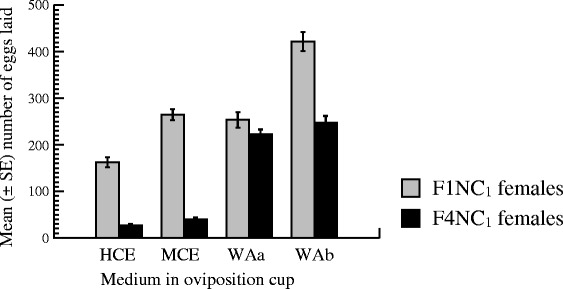
Responses of gravid *Ae. albopictus* F1NC_1_ and F4NC_1_ females when given a choice of ovipositing in four cups containing coffee media at different levels with water ovicups.

When there was an equal chance for oviposition in two cups containing coffee extracts and two others containing water, F1 females deposited eggs in all four cups. Of the 4414 eggs laid, 14.74% (651/4414) were found in cups containing HCE, 24% (1059/4414) in cups containing MCE, 23% (1015/4414) in cups containing WAa, and 38.26% (1689/4414) in WAb cups. Although, F1 females tended to oviposit preferentially in cups containing water compared to those with HCE, there were no significant differences in oviposition responses between the different media (ANOVA = 3.073, DF = 3, P = 0.069). Similar to the F1s, F4 females laid eggs in all four available cups, but egg deposition varied significantly with medium type (ANOVA = 9.853, DF = 3, P = 0.001); a total of 2144 eggs were laid, of which 4.94% (106/2144), 7.37% (158/2144), 41.51% (890/2144), and 46.17% (990/2144) were deposited in HCE, MCE, WAa, and WAb cups, respectively. Tukey’s pairwise comparison revealed that the mean number of eggs laid in cups containing HCE was similar to that in MCE cups (Tukey HSD, P = 0.994), which was significantly lower than those in cups containing WAa (Tukey HSD, P = 0.021) and WAb (Tukey HSD, P = 0.009). There was no significant difference in oviposition response between the cups containing water (Tukey HSD, P = 0.964) (Figure 5).

### Comparison of oviposition responses in relation to history of coffee exposure in the larval stage

In HCE cups, oviposition responses varied significantly with female type (ANOVA = 8.049, DF = 2, P = 0.010). The mean number of eggs laid by F1NC_1_ females in HCE cups was significantly higher than those deposited by their F1NC_0_ (Tukey HSD, P = 0.019) and F4NC_1_ (Tukey HSD, P = 0.019) counterparts. The latter two female types (F1NC_0_ and F4NC_1_) deposited eggs in similar numbers (Tukey HSD, P = 0.989). Female type significantly affected oviposition response level in cups containing MCE (ANOVA = 12.875, DF = 2, P = 0.002). The mean number of eggs deposited in cups containing MCE was similar between F1NC_0_ and F4NC_1_ (Tukey HSD, P = 0.859) and markedly lower than that of their F1NC_1_ counterparts. There were no significant differences in mean number of eggs laid in WAa (ANOVA = 1.192, DF = 2, P = 0.347) and WAb (ANOVA = 1.692, DF = 2, P = 0.238) cups between F1NC_0_, F1NC_1_, and F4NC_1_ females (Table 3).

**Table 3:** **Mean (± SE) numbers of eggs laid by different types of** ***Ae. albopictus*** **females in experiments 1, 4, and 5**

**Medium in oviposition cup**	**Female type**		
	**F1NC0**	**F1NC1**	**F4NC1**
HCE	32.00 ± 12.12	162.75 ± 42.72	26.50 ± 15.66
MCE	65.25 ± 31.12	264.75 ± 47.95	39.50 ± 16.52
WAa	346.25 ± 65.29	253.75 ± 66.27	222.50 ± 42.10
WAb	383.00 ± 70. 86	422.25 ± 81.22	247.50 ± 57.31

F1NC_0_: females that had no contact with coffee extract during the larval stage; F1NC_1_: females derived from larvae that survived a 3-hour exposure to HCE during the early stage of development and completed development in LCE medium; F4NC_1_: females derived from larvae as their F2 and F3 larval parents that survived 3-hour exposure to HCE during the early stage of development and achieved development in LCE medium.

## Discussion

Our data show that coffee and its waste have detrimental effects on the oviposition and larval eclosion responses of *Ae. albopictus*. Egg depositions were much lower in the coffee environments. Gravid females retained increased numbers of mature eggs when ovicups with highly concentrated coffee extract were the unique egg laying sites. Egg hatching success was extremely low among eggs that matured on substrates soaked with coffee extracts.

There was a clear relationship between coffee extract concentration and egg deposition activity, with ovicups containing HCE showing lower attractiveness than those containing MCE, which in turn were significantly less attractive to gravid *Ae. albopictus* females than cups containing water. The attractiveness of an oviposition site to gravid mosquito females in competition with other egg deposition sites is influenced by a number of factors. Specifically, physicochemical aspects of the aquatic medium [27], visual cues [74], and likelihood/possibility of adverse effects on larval development [75,76] have been reported to be the major factors affecting egg deposition decision. Santos *et al.* [77] evaluated the oviposition activities of *Ae. aegypti* in relation to infusion concentration and found that females preferred containers with highly concentrated solutions to those with low concentrations of extracts. Choice of egg deposition site is also associated with colour of the oviposition medium. Li and collaborators [78] examined the impacts of various coloured media on oviposition activities of *Culex pipiens pallens*, and found significantly greater egg deposition in dark blue water than distilled water. Oviposition habitat selection by dengue mosquitoes in response to media of plant origin is related to the presence of toxicants. For example, the oviposition activities of *Ae. aegypti* were examined in relation to the presence of four egg deposition sites consisting of water and three different concentrations of CB extract [17]; the results demonstrated a preference for oviposition sites with CB media. Containers with 1CB and 3CB solutions showed greater attractiveness to ovipositing females than water; but those with the 5CB solution were far less attractive to gravid *Ae. aegypti* than water. It was suggested that the high toxicity due to the presence of five CBs deterred females from laying eggs in 5CB containers. In an earlier study [31], maple leaf litter was found to be a more suitable substrate for *Ae. albopictus* development than camphor leaf litter. This was attributed to the rapid decomposition of maple leaves and the associated increased release of labile substances that act as nutritional resources. It was also reported that the maple solution had a darker colour than the camphor solution. In another study, the effects of maple and cinnamon leaf extracts on the oviposition preference of *Ae. albopictus* were examined [18]. The results indicated increased preference for containers containing maple leaf extract solution over those with cinnamon leaf extract or water, which suggested that *Ae. albopictus* females can respond to greater feeding chances for their larvae. In a study to determine the effects of CB extracts on *Ae. albopictus* oviposition activities [32], egg deposition gradually increased with CB concentration, with cups containing the highest level of decomposition (D9CB solution) receiving the highest mean number of eggs. This increased oviposition response was attributed to the presence of leachates that rendered the medium dark brown, a colour that acts as a visual stimulus regarded by females as a good indicator of the presence of food resources and thus suitable for egg deposition. The present oviposition choice study was carried out with three different oviposition media, *i.e*., HCE, MCE, and water. Visually, HCE was dark brown, MCE was light brown-orange, and water was colourless. In all oviposition choice tests (cups: HCE, MCE, WAa, or WAb; cups: HCE, WAa, WAb or WAc; cups: HCEa, HCEb, HCEc, or WAa), *Ae. albopictus* females showed markedly greater rates of oviposition in water (WA) cups than coffee cups (HCE and MCE). Coffee contains many toxic chemicals [49,50]. Therefore, it is likely that *Ae. albopictus* can determine the quality of an egg deposition site with respect to its negative effects on larval development. Taken together with the results reported previously [31], these observations suggest that this mosquito can discriminate between habitats with brownish water with regard to the presence of food resources or toxicity exposure potential.

The hatch rate of *Ae. albopictus* eggs that developed on filter papers saturated with water was nearly 100%, while the unsuccessful hatching rate was high among eggs maintained on coffee-impregnated paper, especially those in HCE tubes (>80%). Many parameters are involved in determining the hatch success of an *Aedes* egg. Specifically, the characteristics of the hatching medium [79,80] and pre-hatching conditions [81,82] have been shown to be major factors influencing larval eclosion. All eggs examined for larval eclosion here were flooded in the same hatching solution [2 mL of 2-day-old tap water added to 2 droplets of dried yeast solution (0.003 g/100 mL)] and volume; therefore, differences in hatching success rates could not be due to differences in hatching medium. For most *Aedes* species, freshly oviposited eggs undergo a darkening process during which the embryo must absorb moisture for successful embryonic development [83,84]. The viability of such eggs depends largely on postoviposition moisture conditions [85,81,18,82]. This water absorption results in an increase in size, and the rate of expansion varies with the source of moisture [86]. There is a close relationship between the nature of the moisture source and the potential for moisture uptake by the embryo. Such links have been well documented in mosquitoes. Rosay [86], working with *Culex tarsalis* and *Aedes nigromaculis* found that maintenance on water paper substrate resulted in longer egg length compared to those kept on paper substrates moistened with oil. In mosquitoes, the concentration of the moisture source has been shown to affect the intensity of embryo water uptake. For example, in *Anopheles* species, maintenance in hypertrophic solution interferes with the lengthening of eggs [87]. In *Ae. aegypti*, eggs lengthened to a lesser extent when maintained on a dry surface in a saturated atmosphere than when kept on a damp surface [88]. The ease of hatching of *Aedes* eggs is dependent on the adequacy of water uptake during development. Indeed, eggs that absorb sufficient water hatch easily and at a greater rate than those that take up less water [89,82]. In the present study, maturation in the HCE environment resulted in a low hatching rate compared to maturation in low-concentration coffee-contaminated environment (MCE filter paper); almost all eggs maintained in the water environment hatched. It is generally assumed that insect eggs, including those of *Ae. albopictus*, lose water during development via osmosis [90,82]. Although we did not determine the osmotic currents between the ovipositional substrates examined here (HCE-, MCE-, and water-imbibed filter papers) and exposed *Ae. albopictus* eggs, the observed differences in hatching responses may have been due to at least two processes. First, the high ionic balance in coffee-soaked oviposition substrates, particularly in the HCE filter papers, may restrict water transfer inside eggs, thereby increasing the probability of non-viability. Second, the low ionic balance in the condensed water-saturated filter papers could allow water passage through the chorion and hence increase the probability of adequate water uptake as well as viability. This latter process is probably more pronounced in HCE-imbibed eggs. The observed differences in hatching rates between the coffee- and water-treated eggs may be explained by differences in enzymatic activities during maturation. In fact, freshly laid eggs of dengue mosquitoes, kept under moist conditions, gradually darken and harden within approximately 2 – 4 hours postoviposition [91,84]. This darkening process is controlled by the activities of DOPA decarboxylase, an enzyme required for the synthesis of *N*-acetyldopamine, a sclerotizing agent [92]. As the egg matures, the DOPA decarboxylase present on the egg chorion since oogenesis is activated, leading to its hardening [91]. This process also involves phenoloxidase, an enzyme responsible for activating the tanning (darkening) pathway in insects [93,94]. Monnerat and colleagues [95] reported a delayed darkening process in *Anopheles albitarsis* eggs immersed in a benserazide solution and credited it to a repressive effect of the solvent on DOPA decarboxylase. Li and Christensen [96] observed a high degree of hatchability of tanned *Ae. aegypti* eggs and increased levels of phenoloxidase. In *Ae. albopictus*, Xue and co-workers [97] observed reduced hatching success of untanned eggs, which they attributed to low accumulation of L-DOPA (L-3,4-dihydroxyphenylalanine). In the present study, we exposed eggs to different media for the same period, which resulted in different hatching responses. Based on these previous reports, it is therefore likely that the phenoloxidase levels were greater in eggs soaked with water, and it is possible that the observed differences in hatching rates between the water and coffee treatments occurred due to the lower levels of DOPA decarboxylase in coffee-treated eggs. This effect of coffee on DOPA decarboxylase level is likely to be more pronounced in eggs that matured in the HCE environment. It is also likely that the presence of coffee interfered with the DOPA decarboxylase activity.

The number of eggs collected from ovaries 5 days after blood feeding was high in *Ae. albopictus* females when cups filled with HCE were the only sites available, compared to when cups containing other solutions were available, indicating that HCE cups were not suitable for egg deposition. Similar observations were reported previously in mosquito vectors, including *Ae. albopictus*. In *Culex pipiens*, the removal of oviposition cups from cages a few days after blood feeding was reported to result in increased egg retention [98]. In *Ae. aegypti*, deprivation of oviposition substrates 7 days after blood meal uptake caused females to retain increased numbers of mature eggs in their ovaries [99]. Bar-Zeev and Ben-Tamar [100] reported that gravid *Ae. aegypti* refused to lay eggs when presented with oviposition sites containing DEET (*N*,*N*-diethyl-3-methylbenzamide). Von Windeguth and workmates [101] reported that containers with carbaryl, propoxur, temephos, and methoxychlor were repellent to gravid *Ae. aegypti* females. Verma [102] reported that synthetic pyrethroids deterred such females by preventing their preoviposition posture. Similarly, Canyon [103] reported a significantly reduced oviposition response of *Ae. aegypti* in containers holding either malathion, temephos, permethrin, methoprene, or *Bacillus thuringiensis israelensis* compared to those containing water. Xue *et al*. [104] examined the effects of water mixed with acetone and water mixed with DEET on oviposition behaviour in *Ae. albopictus*, and found increased egg retention and numbers of untanned eggs. In mosquitoes, egg retention has been shown to have detrimental effects on general fitness [105]. In *Ae. albopictus*, keeping mature eggs inside the ovaries results in reduced fecundity and fertility [104]. Xue and collaborators also [97] assessed the effects of piperidine compounds and DEET on the oviposition behaviours of *Ae. albopictus*. They tested these products at a concentration of 0.1% and observed >50% oviposition deterrence against laboratory and field populations for 13 and 21 days, respectively. They suggested that these insect repellents may be useful as oviposition deterring agents for *Ae. albopictus*. In the present study, 55.86% (576/1031) of the eggs were retained by females when HCE cups were the only oviposition sites available. This level of oviposition deterrence was high compared to that reported by Xue *et al*. [97].

In the generational oviposition preference study, both F1NC_0_ and F4NC_1_ females demonstrated increased preference for ovicups containing water and markedly ignored the cups containing coffee (HCE and MCE). F1NC_1_ females were weakly attracted to water cups compared to F1NC_0_ and F4NC_1_; rather, they tended to prefer cups containing coffee extracts, particularly MCE. Coffee contains large numbers of chemicals [106], including alkaloids (e.g., trigonelline), phenolic compounds (e.g., chlorogenic acid), other acids (e.g., quinic, malic, and citric acids), and methylxanthines (e.g., caffeine) [107]. Alkaloids act as feeding deterrents and are lethal to a number of insects [61]. Chlorogenic acid has been shown to reduce the bioavailability of amino acids and to decrease nutrient assimilation in lepidopteran larvae [108], coleopterans [109], cicadellids [110], and small sap-sucking insects [50]. Quinic acid is a phytochemical that contributes to the acidic taste of coffee [111]. Methylxanthines hinder insect feeding and are pesticidal at concentrations known to occur in plants [60], and inhibit the feeding activity of coffee borer beetles [62]. Caffeine, one of the major active components of coffee [112], is renowned for improving memory and the speed with which brains process information in humans [113]. As in humans, caffeine boosts memory in bees, enhancing their long-term memory—honeybees fed caffeinated nectar were three times more likely to remember a flower’s scent than bees fed sugar alone [114]. Bees and mosquitoes are dipterans and have antennae with sensors that they tune to chemicals to find plant or human hosts to feed their eggs [115]. Bees can learn to associate visual and odour cues with the food resource, and the egg-laying behaviour of mosquitoes is known to be influenced by odours [115,20]. To test the effects of caffeine on bee learning and memory formation, Wright *et al*. [114] used appetitive olfactory conditioning [116] to train individual bees to associate floral scent with sucrose and different concentrations of caffeine. Briefly, conditioning consisted of short-term contact of the antennae with the conditioned stimulus (caffeine) and the unconditioned stimulus (sucrose solution). The amounts of caffeine tested ranged from 0.05 to 0.35 mM. They reported that low doses of caffeine in reward had a weak effect on the rate of learning and a profound effect on long-term memory. Referring to Gilbert *et al*. [117] (218 / μg/mL in 170 mL of roasted coffee solution), the caffeine concentrations used in the present study (1.17 – 4.7 grams/150 mL) were higher than those tested by Wright *et al*. [114]. In addition, the frequency and intensity of contacts between *Ae. albopictus* larvae and coffee in the present study were higher than those applied by Wright and colleagues [114]—the caffeine was presented for about 5 s and sugar lick was allowed for about 4 s; a single training trial lasted 28 s. In the present study, larvae had contact with the HCE medium for 3 hours and they developed in MCE medium until pupation. By doing so, we obtained two different patterns of oviposition preference for F1NC_1_ and F1NC_0_ females. The first females derived from larvae that had 3-hour contact with HCE and developed as larvae in the MCE environment were highly attracted to cups containing coffee extracts, whereas the second females that had no contact with HCE and developed as larvae in a water environment avoided laying eggs in either HCE or MCE cups. As in many mosquito species, the larval antennae of dengue vectors possess many odorant receptors [118], sensory cones (multiparous sensillae) and peg organs (taste sensillae) [119]. Due to permanent contact with coffee media, *Ae. albopictus* larvae are constantly exposed to the coffee scent. This increased exposure to the coffee will therefore allow them to develop familiarity with its smell. Based on the reports mentioned above, it is tempting to suggest that F1NC_1_ learned to relate coffee odour with suitability for completion of larval development, explaining why they substantially oviposited in coffee cups, especially in those holding MCE. In support of this suggestion, it has been reported that mosquitoes reared in an odourous environment that is typically repellent associate that smell with “suitable breeding sites.” When given a choice, they preferentially oviposit in such breeding sites; because they developed successfully in such sites, they consider it as suitable for their offspring [115]. The observed low egg laying responses in coffee cups by F4NC_1_ females suggested that the trait “coffee odour is associated with suitable development” did not continue into the fourth generation.

## Conclusions

Coffee, which is the world’s most widely traded commodity after petroleum [37], is the most popular beverage in the world with over two billion cups consumed yearly [120]. Preparation of coffee drinks consists of the roasting of coffee beans, grinding of roasted beans, mixing with hot water, and separation of the liquid coffee from the used ground particles, or bags, which are usually discarded. Therefore, huge amounts of coffee product waste end up as refuse worldwide [37]. In Hong Kong, Starbucks annually produces about 5000 tons of used coffee grounds that are disposed of in landfills [39]. In the UK, several million tons of coffee grounds are discarded as waste every year [38]. In North America, almost two million metric tons of spent coffee grounds are generated annually and are either put into landfills or processed at waste facilities [121]. The chemical richness and diversity of coffee in relation to processing have been well established. This commodity contains thousands of chemicals [106,44,113], including alkaloids (e.g., trigonelline), phenolic compounds (e.g., chlorogenic acid), other acids (e.g., quinic, malic, and citric acids), and methylxanthines (e.g., caffeine) [107]. As outlined above, these compounds have a number of effects on insects [61,50,110,109,108,60,62]. Caffeine, the key ingredient in coffee [122] causes genetic disorders in hamsters [123]. Maternal caffeine exposure in pregnant mice causes cognitive deficits in the offspring [124]. Caffeine inhibits enzymes in the nervous systems of herbivorous insects, triggering paralysis, death, and reproductive deficits [125]. It also poses a serious threat to aquatic organisms [122]. Some coffee products have detrimental effects on biomass and reproduction in cattle and poultry farms [53]. Coffee grounds were shown to cause diuresis and renal, urethral, and bladder irritation in cattle [126]. Although several recycling options are available to minimize the quantity of waste generated and its impact on animal health and the environment [127,128], disposal of this type of waste still represents a serious challenge worldwide. For example, landfilling of coffee grounds associated with food waste produces huge amount of methane [129], a gas that contributes to climate change [130]. With its growing production and consumption [131,120], huge amounts of coffee byproducts and waste as chemical resources and contaminants will accumulate in the environment. As the adverse environmental impacts of coffee and coffee-derived waste materials are also likely to increase, substitutes or auxiliary disposal strategies are therefore needed. The present study was performed to determine the impacts of fresh and used coffee on the ovipositional behaviours and responses as well as larval eclosion of *Ae. albopictus*, with respect to their possible use in control methods. The results indicated that the presence of coffee extracts in artificial containers as breeding sites prevent females from laying substantial numbers of eggs. In addition, the presence of only oviposition sites with HCE induced many females to retain most of their mature eggs within ovaries. In particular, this study indicated that there is a deleterious impact on the embryonic development of *Ae. albopictus*; eggs that matured in either HCE or MCE environments were less viable than their counterparts that were maintained in a water-moistened environment. Finally, this study showed that females that developed successfully as larvae in a coffee environment tended to heavily oviposit in container habitats holding coffee. These results illustrate the potential of coffee extracts to assist in dengue vector control, acting both as an uninviting signal to gravid *Ae. albopictus* females and as a barrier to embryogenesis. These attributes suggest that coffee and its waste may be useful in developing potent, low-cost, and bio-rational mosquito control strategies. More importantly, turning coffee waste into an alternate control tool against mosquito vectors may represent a viable solution to the coffee-related pollution problem.
